# Effects of Protein Components on the Chemical Composition and Sensory Properties of Millet Huangjiu (Chinese Millet Wine)

**DOI:** 10.3390/foods12071458

**Published:** 2023-03-29

**Authors:** Chenguang Zhou, Yaojie Zhou, Tianrui Liu, Bin Li, Yuqian Hu, Xiaodong Zhai, Min Zuo, Siyao Liu, Zhen Yang

**Affiliations:** 1 Agricultural Product Processing and Storage Lab, School of Food and Biological Engineering, Jiangsu University, Zhenjiang 212013, China; 2 National Engineering Laboratory for Agri-Product Quality Traceability, Beijing Technology and Business University, Beijing 100048, China; 3 School of Pharmacy, Jiangsu University, Zhenjiang 212013, China; 4 Key Laboratory of Nuclear Agricultural Sciences of Ministry of Agriculture and Zhejiang Province, Institute of Nuclear Agricultural Sciences, Zhejiang University, Hangzhou 310058, China

**Keywords:** millet Huangjiu, protein, free amino acids, HS-SPME-GC/MS, sensory properties

## Abstract

Millet Huangjiu is a national alcoholic beverage in China. The quality of Chinese millet Huangjiu is significantly influenced by the protein components in the raw materials of millet. Therefore, in this study, the impact of different protein components on the quality of millet Huangjiu was investigated by adding exogenous proteins glutelin and albumin either individually or in combination. The study commenced with the determination of the oenological parameters of different millet Huangjiu samples, followed by the assessment of free amino acids and organic acids. In addition, the volatile profiles of millet Huangjiu were characterized by employing HS-SPME-GC/MS. Finally, a sensory evaluation was conducted to evaluate the overall aroma profiles of millet Huangjiu. The results showed that adding glutelin significantly increased the contents of total soluble solids, amino acid nitrogen, and ethanol in millet Huangjiu by 32.2%, 41.5%, and 17.7%, respectively. Furthermore, the fortification of the fermentation substrate with glutelin protein was found to significantly enhance the umami (aspartic and glutamic acids) and sweet-tasting (alanine and proline) amino acids in the final product. Gas chromatography-quadrupole mass spectrometry coupled with multivariate statistical analysis revealed distinct impacts of protein composition on the volatile organic compound (VOC) profiles of millet Huangjiu. Excessive glutelin led to an over-accumulation of alcohol aroma, while the addition of albumin protein proved to be a viable approach for enhancing the ester and fruity fragrances. Sensory analysis suggested that the proper amount of protein fortification using a Glu + Alb combination could enhance the sensory attributes of millet Huangjiu while maintaining its unique flavor characteristics. These findings suggest that reasonable adjustment of the glutelin and albumin contents in millet could effectively regulate the chemical composition and improve the sensory quality of millet Huangjiu.

## 1. Introduction

Huangjiu is a Chinese rice wine with a history of over 2500 years. It is produced from glutinous rice, corn, or millet using a multi-step process that involves cooking, saccharifying, fermenting, pressing, filtering, frying, and blending [1]. Huangjiu can be classified into two types based on the production region, namely, Southern Huangjiu from Southern China and Northern Huangjiu from Northern China. The former is typically produced from glutinous rice, whereas the latter is produced from millet [2]. Compared to rice (4.3~7.0%), millet contains a higher concentration of crude protein (11.4~13.7%), which makes it nutritionally valuable and offers unique functional properties for food development and application. Millet has been used in various products, such as yogurts, breads, and supplementary foods for infants, and its high protein content renders it an optimal choice for Northern Huangjiu fermentation [3]. The use of millet in Northern Huangjiu production may enhance its nutritional value and provide distinct functional properties in the final product [4]. Chinese millet Huangjiu is typically characterized by its light color, low alcohol content (11~13%), mellow taste, and smooth mouthfeel. Based on data from the China Alcoholic Drinks Association, the consumption of millet Huangjiu in China has exhibited a steady upward trend in recent years. Specifically, in 2020, the annual sales volume of millet Huangjiu in China was reported to have reached 0.43 billion liters.

Several studies have shown that the flavor of millet Huangjiu is influenced by the raw materials, particularly the protein profiles of millet [2]. Enzymes secreted by fermented bacteria degrade millet proteins and form short peptides and free amino acids. Besides affecting yeast growth, amino acids also affect the quality of Huangjiu, e.g., higher levels of alcohols and esters. Researchers found that during grape wine fermentation processes, nitrogen utilization was positively correlated with total aromatic substance content [5]. Moreover, a positive correlation was found between the consumption of amino acids (e.g., tyrosine, lysine, and leucine) and the formation of alcohols and acetate esters [5]. A previous study indicated that Japanese sake brewed with added protein exhibited a different amino acid composition compared to the control group. Additionally, sensory analysis revealed that the addition of prolamin negatively affected the flavor quality of sake, probably because prolamin is poorly digested during fermentation, resulting in a prolonged fermentation time. Contrary to this, glutelin could be easily digestible into amino acids and oligopeptides. As a result, glutelin may significantly enhance sake’s nutritional content and taste. It was confirmed through a clarification experiment that adding glutelin to white wine enhanced the clarity and preserved the aromatic compounds, as well as improved the flavor [6]. Similarly, due to the excellent thermal stability and surface properties (e.g., emulsifiability, foamability, and solubility) within a broad range of pH levels, from 3.0 to 8.0, albumin may also serve as a food ingredient to develop high-quality wine [7].

The quality of Huangjiu brewed using different raw materials varies greatly. Limited information is only available on the glutinous rice Huangjiu [8,9], whereas the effects of millet protein components on the volatiles and physicochemical properties of millet Huangjiu have not been thoroughly studied. Accordingly, the addition of exogenous glutelin and albumin was used to modify the protein composition of the brewing raw materials, aiming to examine how different protein components affect the flavor and quality of millet Huangjiu. The goal of the current study was to establish a correlation between the protein components of brewing materials and the overall quality of millet Huangjiu, as well as to offer some guidelines regarding millet selection for aroma enhancement in Huangjiu brewing.

## 2. Materials and Methods

### 2.1. Chemicals

The internal standard 2-octanol (purity ≥ 99%) was supplied by Macklin Inc. (Shanghai, China). The alkane standard mixtures (C7 to C40 straight-chain alkanes) were obtained from Sigma-Aldrich (Steinheim, Germany). Exogenous commercial proteins (purity ≥ 98%) were purchased from Shannxi Pioneer Biotech Co., Ltd. (Shannxi, China). Other chemicals, including NaOH, sulfosalicylic acid, acetic acid, and KH_2_PO_4_, were purchased from Sinopharm Chemical Reagent (Shanghai, China).

### 2.2. Preparation of Millet Huangjiu

The foxtail millet Jingu, a commonly cultivated variety in Northwest China, served as the raw material for the production of millet Huangjiu in this study. The millet Huangjiu samples were prepared in accordance with the traditional Huangjiu preparation methods used in Northern China. In brief, 500 g of millet was soaked at 25 °C for 20 h, steamed for 30 min, and then cooled to 30 °C. Yeast strains were pre-cultured in yeast extract-peptone-dextrose (YPD) medium for 24 h at 30 °C and then cultured for an additional 24 h with the addition of 100 mL of YPD medium. Following centrifugation, the yeast cells were incorporated with 1 L of water, 50 g of naturally inoculated wheat Qu (comprising molds, bacteria, and strains), 500 g of steamed millet, and exogenous commercial proteins (purity ≥ 98%) within a pottery jar.

The protein content was estimated at approximately 12%, yielding around 60 g of endogenous proteins (from 500 g raw materials) per jar. Typically, glutelin and albumin proteins constitute approximately 20% and 5% of the total protein content in millet, respectively. To facilitate optimal fermentation conditions, equal amounts of exogenous glutelin and albumin (12 g and 3 g, respectively) were added to the fermentation substrate. Additionally, double amounts of these proteins (24 g and 6 g, respectively) were also added as an alternative approach. According to the types and the amounts of proteins added, the millet Huangjiu samples were categorized into six groups: the control group (no exogenous protein added), the Glu-1 group (12 g of glutelin), the Glu-2 group (24 g of glutelin), the Alb-1 group (3 g of albumin), the Alb-2 group (6 g of albumin), and the Glu + Alb group (12 g of glutelin and 3 g of albumin). Each mixture was fermented at 30 °C for 7 days followed by 27 days at 15 °C, with three replicates for each fermentation. After sample preparation of millet Huangjiu, fermentation mash was collected for the analysis of physicochemical properties, organic acids, free amino acids, and volatile organic compounds (VOCs).

### 2.3. Oenological Parameter Analyses of Millet Huangjiu

The total soluble solids in the millet Huangjiu samples were quantified in Brix (°Bx) using a refractometer. Total sugar content was analyzed according to the AOAC 968.28 method (Association of Official Analytical Chemists, 2002) based on the Lane–Eynon method. Titratable acidity was determined by titrating the samples with 0.01 mol/L NaOH until the pH reached 8.2, with phenolphthalein as the indicator. The ethanol content in the millet Huangjiu was measured using a handheld density meter (DMA 35 Basic, Anton Paar, Austria). All chemical analyses were conducted in triplicate.

### 2.4. Analysis of Free Amino Acids

The millet Huangjiu samples were first pre-filtered and then subjected to precipitation for 2 h at 4 °C using 10% sulfosalicylic acid. Each mixture was subsequently centrifuged at 12,000× *g* for 20 min to eliminate large peptides. To determine the amino acid composition and contents, a 20 μL aliquot of the supernatant was injected into a Hitachi L-8900 automatic amino acid analyzer (Hitachi, Tokyo, Japan). By calibration with standard amino acids, the free amino acid contents in the millet Huangjiu samples were calculated.

### 2.5. Analysis of Organic Acids

After subjecting 5 mL of millet Huangjiu to centrifugation at 10,000× *g* for 10 min, the resulting mixture was filtered through a 0.45 μm microporous membrane. An HPLC analysis was performed to determine organic acids. The separation was conducted on an Agilent 1260 Infinity II system with a 250 × 4.6 mm and 5 μm Ultisil XB-C18 column, following the chromatographic conditions described by Yan et al. [10]. The column temperature was maintained at 30 °C, and a mobile phase consisting of pH 2.3 phosphate buffer (0.01 mol/L KH_2_PO_4_) adjusted with a 5% (*w*/*v*) acetic acid solution was used. The flow rate was set to 0.8 mL/min, and detection was carried out at a wavelength of 210 nm. Organic acid quantification of the samples was performed using an external standard method. The calibration curve equations for each organic acid were as follows: oxalic acid: y = 0.0026x − 0.0034 (R^2^ = 0.9993); tartaric acid: y = 0.0034x + 0.042 (R^2^ = 0.9998); pyruvic acid: y = 0.0018x + 0.0076 (R^2^ = 0.9987); malic acid: y = 0.0032x + 0.0017 (R^2^ = 0.9991); lactic acid: y = 0.0507x + 0.0233 (R^2^ = 0.9986); citric acid: y = 0.0198x − 0.0411 (R^2^ = 0.9995); succinic acid: y = 0.0746x + 0.0347 (R^2^ = 0.9994); acetic acid: y = 0.0269x − 0.0163 (R^2^ = 0.9993); and fumaric acid: y = 0.0027x + 0.0015 (R^2^ = 0.9971). The linear ranges of oxalic acid, tartaric acid, pyruvic acid, malic acid, and fumaric acid were 5.0–500 mg/L. For citric acid and acetic acid, the linear range was 0.05–5 g/L, and for lactic acid and succinic acid it was 0.10–10 g/L.

### 2.6. Analysis of Volatiles in Millet Huangjiu

The flavor compounds of millet Huangjiu were analyzed by solid-phase microextraction (SPME) and gas chromatography-mass spectrometry (GC-MS). For this, a mixture of 2.0 g of millet Huangjiu and 0.5 g of NaCl, along with 20 μL of internal standard (IS) 2-octanol (4.572 μg/mL), was placed in a 20 mL headspace glass vial. The mixture was subjected to 50 °C water bath equilibrium for 20 min. After being equilibrated in a 50 °C water bath for 20 min, a 50/30 mm DVB/CAR/PDMS SPME fiber (Superco, Bellefonte, PA, USA) was introduced into the headspace bottle and allowed to absorb the volatiles for 30 min under continuous heating at 50 °C and agitation. The desorption of volatile compounds enriched in the fiber coating was conducted for 5 min at 250 °C in the GC inlet. The volatile substances were analyzed using a GCMS-TQ8040 instrument (Shimadzu, Kyoto, Japan) equipped with a DB-WAX capillary column (30 m × 0.25 mm × 0.25 μm; Agilent Technologies, Santa Clara, CA, USA). The analysis of volatiles was conducted via GC-MS according to the method reported by Yan et al. [10]. In addition to comparing the NIST 17 mass spectral library, the Kováts retention indexes (RIs) of the extracted volatile compounds were calculated using C7-C40 n-alkanes and verified by reference to the RIs reported in the literature and in online libraries. The concentrations of volatiles were determined using semi-quantitative analysis, referring to the internal standard.

### 2.7. Sensory Evaluation

Ten panelists (5 males and 5 females), aged 22–30, who possessed prior experience in olfactory experiments and quantitative descriptive analysis, were recruited from our laboratory at Jiangsu University. The panelists underwent a preliminary training session, in which they were introduced to standard reference samples of each attribute and were asked to identify them correctly. This training process was repeated until all panelists accurately identified each standard. Following preliminary experiment discussion and referencing relevant studies in the literature [11,12,13], six sensory attributes were selected for the formal evaluation, including alcoholic aroma (referred to as 3-methyl-1-butanol aroma), caramel aroma (caramel), sour aroma (acetic acid), ester aroma (ethyl acetate), fruit aroma (ethyl isovalerate), and Qu aroma (Daqu fermentation starter).

The sensory evaluation of millet Huangjiu involved dispensing 30 mL of each sample into covered, odorless glass cups, which were then randomly distributed. A quantitative sensory evaluation was conducted using a 10-point interval scale for each attribute, with scores ranging from 0 (imperceptible) to 9 (extremely strong). The scores for each attribute were averaged across all 10 panelists, and the analysis was performed in triplicate to ensure accuracy and consistency.

### 2.8. Statistical Analysis

The experiments were carried out in triplicate, and the results are presented as mean values ± standard deviations. Statistical analysis was conducted using ANOVA with Duncan’s multiple range tests, utilizing IBM SPSS Statistics 21 (SPSS Inc., Chicago, IL, USA). For multivariate analysis, the data matrix underwent log transformation and Pareto scaling pretreatment before being imported into MetaboAnalyst for principal component analysis (PCA).

## 3. Results and Discussion

### 3.1. The Oenological Parameters of Millet Huangjiu

The present study investigated the effects of exogenous proteins on various quality parameters of millet Huangjiu. Table 1 summarizes the changes in total soluble solids, reducing sugar content, alcohol content, and amino acid nitrogen content in the produced Huangjiu samples. The control group showed the lowest total soluble solids content (11.8 ± 0.19 °Bx), while the Glu-2 group, with the highest amount of protein addition, exhibited the highest total soluble solids content (15.6 ± 0.21 °Bx). Notably, the amount of protein added before fermentation was also positively correlated with amino acid nitrogen concentrations (Table 1). However, the effect of protein components on reducing sugar content was found to be non-significant, though these values were still within the same range as concentrations reported in previous studies on traditional millet Huangjiu [10,12]. Similar findings were also reported in rice Huangjiu and sake [8,9].

A notable correlation exists between the concentration of ethanol in millet Huangjiu and the glutelin content of its fermentation substrate (Table 1). Interestingly, the ethanol contents in Huangjiu samples were increased by 8.2%, 7.3%, and 17.7% in the Glu-1, Glu + Alb, and Glu-2 groups, respectively. On the contrary, no significant differences were observed between the control group and the albumin-added groups Alb-1 and Alb-2. During the production process, millet starch is hydrolyzed into dextrin by the enzyme α-amylase and subsequently into glucose by glucoamylase [14]. Yeasts then ferment the glucose to produce ethanol, the primary alcohol in Huangjiu. A recent study investigated the role of glutelin, a protein found in millet, in the fermentation process of Huangjiu. The results showed that higher levels of glutelin in the fermenting materials resulted in a more rapid conversion of sugar to alcohol than in the control group [15]. This suggests that glutelin may promote yeast growth and enhance the utilization of sugar for ethanol production. The concentration of ethanol can be used as a reliable indicator of the fermentation status of Huangjiu during the production process. The findings of this study support the hypothesis that glutelin plays a significant role in the completion of millet Huangjiu fermentation and its quality.

### 3.2. Changes in the Free Amino Acid Fraction of Millet Huangjiu

Millet Huangjiu is renowned for its distinctive taste and aroma, which can be attributed, in part, to its high amino acid content. Amino acids are not only essential for yeast metabolism and growth during the fermentation process, but also serve as precursors for a range of flavor and aroma compounds [16]. Analysis of millet Huangjiu using a high-speed amino acid analyzer revealed the presence of 17 different free amino acids (Table 2). Among these, Glu was found to be the most abundant in the control group, with a concentration of 268.2 mg/L, followed by Ala and Pro, with levels of 195.6 mg/L and 155.5 mg/L, respectively. In contrast, the lowest concentration of free amino acids was detected for His, with only 25.8 mg/L observed in the control group.

Table 2 revealed that the 17 identified amino acids in millet Huangjiu were classified based on their taste presentation characteristics into umami, sweet, and bitter tasting. The results showed that Glu, Leu, Phe, Gly, Ala, and Pro were the most abundant amino acids, with concentrations exceeding 100 mg/L, accounting for over 60% of the total amino acid contents. The predominance of these amino acids in Huangjiu may contribute to its unique taste and flavor. The production of free amino acids during fermentation occurs mainly through enzymatic degradation of proteins in the raw material by microorganisms that secrete proteases, as well as by intracellular proteases following the autolysis of microorganisms [17]. This process can lead to the release of amino acids from proteins, resulting in their accumulation in the fermented product [18].

The results indicate that the Glu-1, Glu-2, Alb-1, Alb-2, and Glu + Alb groups showed a significant increase in total amino acid content, ranging from 7.2% to 49.9%, with the highest level reaching up to 2567.4 mg/L in the Glu-2 group. Specifically, the umami amino acids Asp and Glu exhibited the most pronounced increase, with contents rising to 135.4 mg/L and 332.3 mg/L, respectively. In contrast, the addition of albumin (Alb-1 and Alb-2) showed no effect on the umami amino acid contents. The most abundant sweet-tasting amino acid, Ala, was almost twice as high in the Glu-1 compared to the control group. Interestingly, the extra addition of glutelin (Glu-2) did not further increase the concentrations of Ala in millet Huangjiu, indicating a saturation effect. In contrast, the levels of the sweet amino acids Ser, Gly, and Thr showed a dissimilar variation tendency, with the addition of glutelin having no effect on their contents, whereas a slight increase in their contents, ranging from 9.9% to 21.1%, was observed in the Alb-1, Alb-2, and Glu + Alb groups. These results demonstrate that variation in the contents of sweet amino acids in millet Huangjiu depends heavily on the type and amount of protein added to the fermentation substrate.

The addition of albumin to millet Huangjiu resulted in minor changes in the levels of bitter-tasting amino acids, including Arg, Val, Ile, and Leu. In contrast, His, Tyr, Met, Cys, and Phe exhibited at least a two-fold increase in the millet Huangjiu sample from the Glu-2 group. Previous research has suggested that the production of bitter peptides in sake is highly dependent on the glutelin content of rice grains [8], highlighting the significance of protein metabolism in shaping the flavor profile of fermented beverages. The development of bitterness in Huangjiu is thought to arise from an imbalance in protein hydrolysis and peptide hydrolysis, emphasizing the importance of carefully balancing the glutelin contents in raw materials to optimize wine quality [4,6,9]. Taken together, these findings suggest that carefully selecting the appropriate protein type and amount during the fermentation process is essential to achieving the desired taste profile in millet Huangjiu.

### 3.3. Changes in Organic Acids in Millet Huangjiu

**Figure 1:**
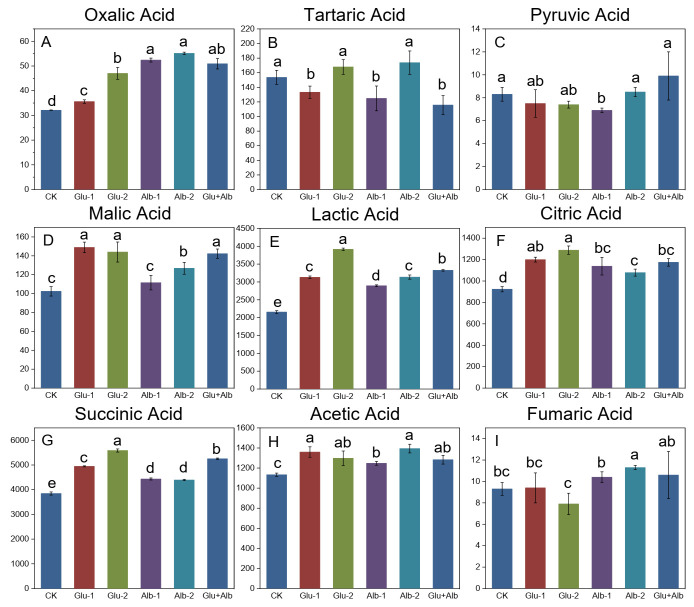
Concentrations of organic acids in millet Huangjiu upon addition of exogenous proteins. Different letters indicate statistically significant differences (*p* < 0.05). (**A**) oxalic acid, (**B**) tartaric acid, (**C**) pyruvic acid, (**D**) malic acid, (**E**) lactic acid, (**F**) citric acid, (**G**) succinic acid, (**H**) acetic acid, and (**I**) fumaric acid.

Organic acids play a crucial role in the formation of the flavor and physiological properties of millet Huangjiu. Their presence in appropriate amounts contributes to a harmonized and stabilized taste and aroma, thereby rendering the Huangjiu more palatable [19]. As depicted in Figure 1, the identified organic acids included oxalic acid, tartaric acid, pyruvic acid, malic acid, lactic acid, citric acid, succinic acid, acetic acid, and fumaric acid. The predominant organic acids detected in the control group were succinic acid (45.9%), lactic acid (25.8%), acetic acid (13.6%), and citric acid (11.1%), which accounted for 96.4% of the total organic acid content. This finding is comparable to the results reported by Yi et al. [10]. Pyruvic acid and fumaric acid exhibited the lowest contents, fluctuating around 8–9 mg/L. The concentrations of pyruvic acid and fumaric acid were observed to be relatively low, with fluctuations within the range of 8–9 mg/L. Tartaric acid and malic acid were present in moderate concentrations, at 153.6 mg/L and 102.4 mg/L, respectively.

The impacts of glutelin and albumin addition on the contents of the primary organic acids, lactic acid and succinic acid, are illustrated in Figure 1E,G. The results showed that the levels of lactic acid and succinic acid were significantly increased after the addition of proteins prior to the fermentation process. The extent of these increases was positively correlated with the amount of proteins added, with the highest levels recorded in the Glu-2 group at 3914.0 mg/L and 5585.2 mg/L, respectively. Interestingly, the impact of glutelin and albumin on the production of lactic acid and succinic acid was not uniform. The addition of glutelin resulted in a more significant increase in both amino acids compared to albumin, with higher concentrations recorded in the Glu-1 group at 3130.1 mg/L and 4943.7 mg/L, respectively, compared to 2893.8 mg/L and 4433.3 mg/L in the Alb-1 group. A similar disparity was also noted between the Glu-2 and Alb-2 groups. Succinic acid is not only an intermediate metabolite in the tricarboxylic acid cycle (TCA), but also a precursor to other metabolic pathways, with a distinctive umami taste [20]. These results suggest that adding an appropriate amount of glutelin and albumin to the fermentation substrate can enhance the umami taste in millet Huangjiu, thereby mitigating the pungent sour taste resulting from an increase in acetic acid levels (Figure 1H) [21].

In contrast to the marked variations observed in the contents of succinic and citric acids, the levels of minor amino acid components, namely, pyruvic acid and fumaric acid, remained unaltered upon treatment with glutelin and albumin (Figure 1C,I). It is worth noting that pyruvic and fumaric acids are intermediate metabolites of glycolysis and the citric acid cycle [14]. Under aerobic conditions, pyruvic acid acts as a major energy source for microbial cells via the citric acid cycle. However, under anaerobic conditions, such as alcoholic fermentation, pyruvic acid is converted to ethanol [22]. Since pyruvic acid is not a final product of the fermentation process, the low accumulation of pyruvic acid in the millet Huangjiu samples indicates that the yeast cells have sufficient glycolytic and citric acid cycle activities [18]. This finding suggests that the fortification of protein content in the fermentation substrate with glutelin and albumin has little adverse impact on the Huangjiu production process.

### 3.4. Changes in VOCs in Millet Huangjiu

The volatile components of millet Huangjiu samples with varying protein additions were extracted using HS-SPME and analyzed via GC-MS. The volatiles were then identified based on their mass spectrum and KI values. The aroma profile of millet Huangjiu is a complex blend of various flavor components. A total of 56 volatile compounds were identified in the millet Huangjiu samples, comprising 22 esters, 13 alcohols, 10 aldehydes, 6 acids, 3 phenolics, and 2 ketones, as shown in Table 3. These results are generally consistent with previous reports on the volatile composition of other millet Huangjiu samples [4,10,12].

**Figure 2:**
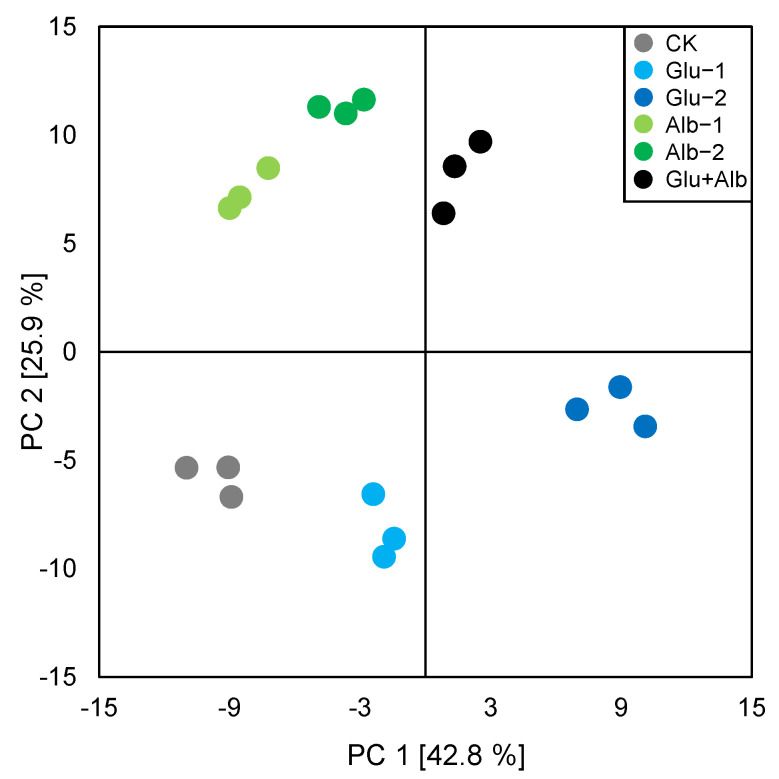
PCA score plot of the volatiles from the different millet Huangjiu samples.

**Figure 3:**
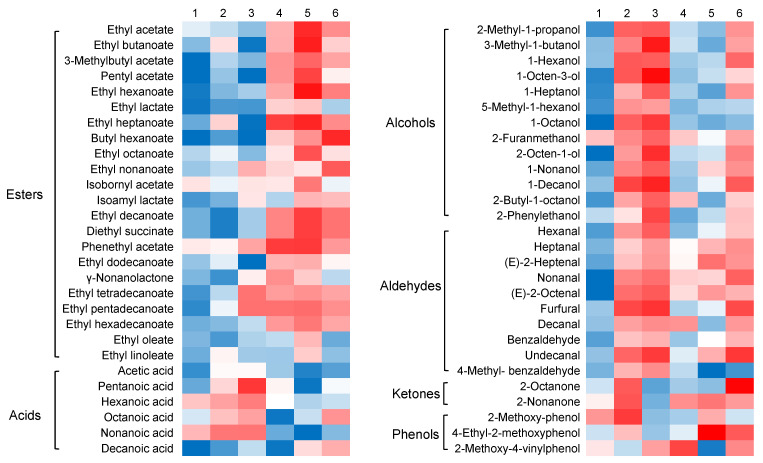
Heat map of the volatiles contributing to the metabolic discriminations among different millet Huangjiu samples.

**Figure 4:**
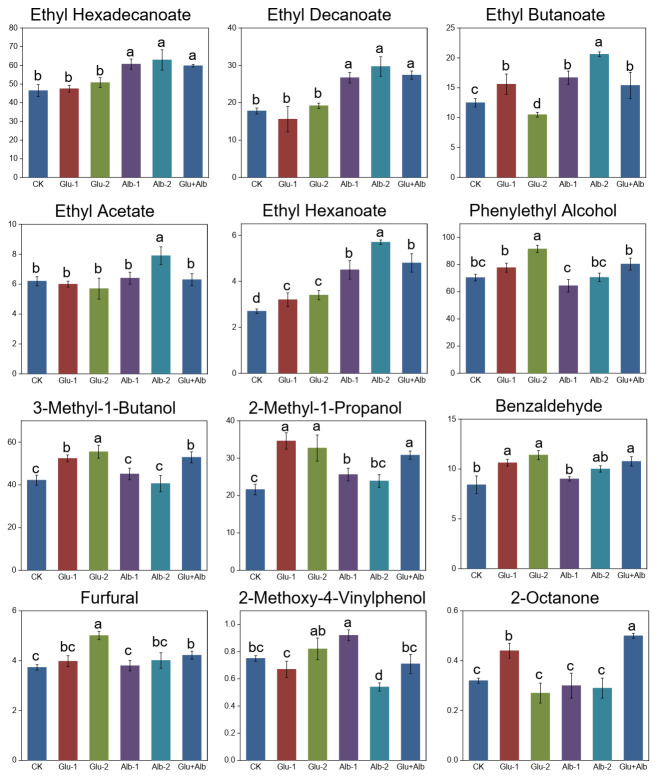
Changes in the concentrations of the predominant volatiles upon addition of exogenous proteins. Different letters indicate statistically significant differences (*p* < 0.05).

The use of principal component analysis (PCA) is a popular unsupervised multivariate statistical technique that reduces the dimensionality of complex multivariable data by integrating a small number of variables, known as principal components. This technique is commonly used to visualize and determine the main sources of variability in a dataset. In this study, PCA was employed to investigate the influence of protein fortification, specifically glutelin and albumin addition, on the volatile profiles of various millet Huangjiu samples. The results displayed in Figure 2 show a clear separation between the millet Huangjiu samples fortified with protein and the control group, indicating significant differences in their volatile profiles. Interestingly, the Glu-1 and Glu-2 samples were separated from the control group along PC1, while the Alb-1 and Alb-2 samples were differentiated from the control group along PC2. These results suggest that the addition of glutelin and albumin may have different impacts on the VOC profiles of millet Huangjiu. Furthermore, the Glu + Alb group was found to cluster more closely with the Alb groups than the Glu groups, indicating a more similar constitution of VOC profiles between the Glu + Alb group and the Alb groups (Figure 2). Esters constituted the highest proportion of volatile compounds in Huangjius, followed by alcohols and aldehydes, collectively accounting for almost 80% of the total volatile contents. To further elucidate the detailed information on the volatiles, a heat map was visualized (Figure 3), and univariate statistical analyses were conducted for the key odorants (Figure 4).

#### 3.4.1. Esters

Esters are the predominant class of compounds contributing to the fruity and floral characteristics of Huangjiu samples [23]. The formation of these compounds is chiefly attributed to esterase-catalyzed reactions of alcohols and acids, which occur due to the metabolism of glucose and amino acids by microorganisms [12]. Ester formation can also occur through non-enzymatic esterification reactions between alcohols and organic acids derived from the substrate during fermentation processes [24]. As indicated in Table 3, ethyl esters are the most abundant form of esters in Huangjiu samples, and they are known for their fruity, sweet, and creamy aroma notes [3]. Key ethyl esters identified in previous studies, including ethyl acetate (fruity, sweet), ethyl octanoate (fruity, mushroom), and ethyl butanoate (fruity, sweet) [25], were also detected in this study, albeit in varying amounts (Figure 3). Of the esters detected, the most abundant ones were ethyl acetate, ethyl butanoate, ethyl hexanoate, ethyl decanoate, and ethyl hexadecanoate, displaying the highest concentrations (Figure 4).

Univariate statistical analysis showed that the concentration of the most predominant ester, ethyl hexadecanoate, was significantly increased from 46.5 mg/L (control group) to 62.9 mg/L (Alb-2 group), with similar increases observed in the Huangjiu samples from the Alb-1 and Glu + Alb groups, but not in the Glu-1 and Glu-2 samples. With the exception of a few minor esters, such as ethyl acetate, most of the major esters showed increased concentrations upon addition of albumin protein (Figure 4). Similar differentiations between the Glu and Alb groups were also observed in the heat map for minor esters, such as ethyl octanoate, γ-nonanolactone, ethyl oleate, and ethyl linoleate (Figure 3). Based on the results, it can be inferred that the inclusion of albumin protein causes a notable increase in the levels of esters, which can potentially influence the fruity and full-bodied aroma characteristics of millet Huangjiu.

#### 3.4.2. Alcohols

Alcohols are the major compounds found in fermented millet wine, which are synthesized via diverse metabolic routes, including amino acid metabolism, reduction of methyl ketones, glycolysis, and degradation of linoleic and linolenic acid [8]. As shown in Table 3, a total of 13 alcohols were identified in millet Huangjiu, with phenylethyl alcohol, 2-methyl-1-propanol, and 3-methyl-1-butanol being the most prevalent, accounting for more than 80% of the total alcohol content detected (Figure 4). This finding is consistent with a previous study by Yan et al. [10]. The volatile component analysis of fermented rice Huangjiu conducted by Yu et al. also showed that 3-methyl-1-butanol and 2-methyl-1-propanol were the predominant alcohols, accounting for over 60% of the total alcohol content [13]. These three fusel alcohols have been demonstrated to be characteristic flavor compounds in fermented alcoholic beverages, contributing to the overall sensory complexity of millet Huangjiu [25,26].

Phenylethyl alcohol, derived from the degradation of phenylalanine [27], was identified as the most abundant fusel alcohol in millet Huangjiu in the present study. This compound occurs naturally in various fermented beverages, including beer, wine, and whisky, and is well-known for its rose-like odor note [28]. In addition, it has also been identified as an odor-active compound in makgeolli [26]. Our study revealed that the addition of glutelin significantly increased the production of phenylethyl alcohol, with concentrations increasing from 70.4 mg/L (control group) to 91.5 mg/L (Glu-2 group) (Figure 4). This increase is likely due to the observed accumulation of phenylalanine (Table 2), which is the precursor of phenylethyl alcohol during the fermentation process. In line with our findings, recent studies have also reported a positive correlation between the levels of phenylethyl alcohol and phenylalanine (Zhou et al.) [29,30].

Likewise, the levels of 3-methyl-1-butanol and 2-methyl-1-propanol in the Glu groups were significantly higher, with increases of approximately 24.2~60.2% compared to the control group (Figure 4). These higher alcohols are known to contribute fruity, winey, and whisky-like notes to the wine [9], thereby enhancing the richness of the taste. In contrast, the levels of these alcohols in the Alb-1 and Alb-2 groups did not differ significantly from those in the control group. These findings suggest that fortifying the fermentation substrate with glutelin protein can enhance the accumulation of amino acids during millet Huangjiu production and significantly increase the production of alcohol odorants, ultimately improving the sensory properties of millet Huangjiu. It should be noted, however, that the concentration of these compounds should be carefully considered during production to avoid undesirable flavors, such as solvent-like aromas. While alcohol volatiles can contribute to the desirable aroma of millet Huangjiu, excessive amounts greater than their critical values can negatively affect its flavor profile [31,32].

#### 3.4.3. Aldehydes

Aldehydes, which are often characterized by their almond and fruit-like notes, play a crucial role in the flavor profile of Huangjiu and have a relatively low odor threshold [33]. These volatile compounds are typically generated via the deamination and decarboxylation of amino acids during the fermentation process [11], and are subsequently accumulated through oxidation reactions of alcohols over the course of aging [34]. In the present study, 10 aldehydes were identified, with benzaldehyde, furfural, and nonanal being the major contributors, accounting for over 70% of the total aldehyde content. As depicted in Figure 4, the millet Huangjiu sample from the Glu-2 group exhibited the most abundant level of benzaldehyde at 11.4 mg/L, which was 35.4% higher than that of the control group. A slightly lesser extent of increase in benzaldehyde content (i.e., 26.2% and 27.9%) was also observed in the Glu-1 and Glu + Alb groups, respectively. According to the findings of Chu and Yaylayan [35], 2-phenylacetaldehyde, which is a Strecker aldehyde originating from phenylalanine, serves as the optimal precursor for benzaldehyde. Consistent with this finding, the present study showed that phenylalanine content increased by 35.1% to 52.3% in the Glu groups compared to the control (Table 3). The changes in other major aldehydes, such as furfural and nonanal, followed a similar trend (Figure 3 and Figure 4). Overall, the findings suggest that the fortification of glutelin in the fermentation substrate can stimulate the production of aromatic aldehydes, enhancing the desirable aroma of the millet Huangjiu product.

### 3.5. Sensory Analysis of Millet Huangjiu Samples

**Figure 5:**
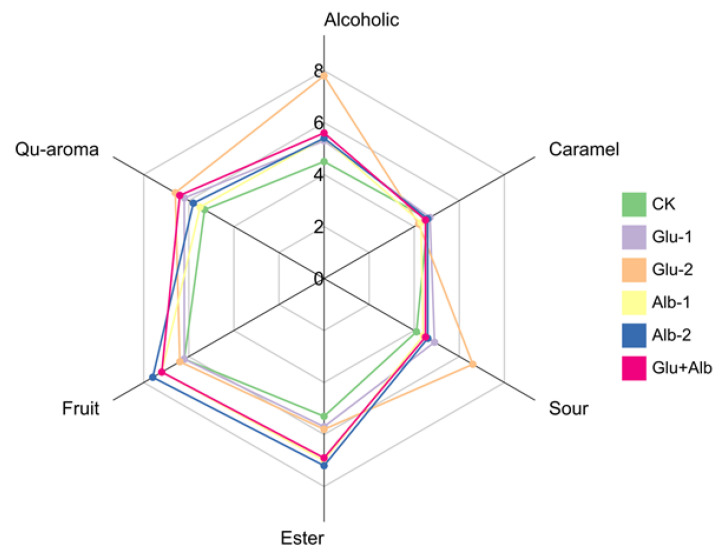
Quantitative descriptive sensory radar chart of the different millet Huangjiu samples. The millet Huangjiu samples were categorized into the control group (CK, no exogenous protein added), Glu-1 group (12 g of glutelin), Glu-2 group (24 g of glutelin), Alb-1 group (3 g of albumin), Alb-2 group (6 g of albumin), and Glu + Alb group (12 g of glutelin and 3 g of albumin).

Sensory evaluation of millet Huangjiu was performed by well-trained panelists to assess the overall aroma profiles. Six aroma attributes, including alcoholic, caramel, sour, ester, fruit, and Qu aromas, were selected for descriptive analysis. The results highlighted distinct differences in aroma profiles across various samples of millet Huangjiu. Figure 5 shows that the Glu-2 group scored the highest for alcohol aroma with a value of 7.8, while the control group had the lowest score (4.5), with the rest of the millet Huangjiu samples falling within a similar range of 5.3~5.6. This result was consistent with the data observed for ethanol contents in Table 1. In comparison with the control group, the protein-fortified samples did not exhibit remarkable changes in the scores for caramel (4.3~4.7) and Qu aromas (5.8~6.6), with the Glu + Alb group achieving the most balanced scores (4.5 for caramel and 6.4 for Qu aroma). In terms of ester aroma intensity, Alb-2 scored the highest (7.2), followed by Alb-1 (7), Glu + Alb (6.9), Glu-2 (5.8), Glu-2 (5.7), and the control (5.3). The finding was further corroborated by the results depicted in the heat map, which exhibited a similar trend in the fluctuations of VOC levels in esters. These findings demonstrate that the addition of albumin protein is a viable approach for augmenting the ester and fruity fragrances of millet Huangjiu. Additionally, the score for the sour attribute suggested that an excess of glutelin (Glu-2) in the fermentation substrate may lead to an unpleasant level of sour aroma (score value of 6.6). Collectively, the appropriate amount of protein fortification employing a Glu + Alb combination demonstrated favorable outcomes in enhancing the sensory attributes of millet Huangjiu, while simultaneously preserving its distinctive flavor characteristics.

## 4. Conclusions

The current study demonstrated that the protein composition of the raw millet material played a significant role in shaping the oenological parameters (i.e., total soluble solids, reducing sugar content, alcohol content, and amino acid nitrogen content) of the final Huangjiu product. Fortifying the fermentation substrate with glutelin was found to significantly increase the umami and sweet-tasting amino acids in the Huangjiu. However, excessive glutelin may lead to an over-accumulation of alcohol, resulting in a solvent-like and sour aroma. In contrast, increasing the albumin component can contribute to the fruity and floral attributes of millet Huangjiu. These findings have practical implications for improving the quality and sensory characteristics of Huangjiu, as well as for the selection criteria for millet used in Huangjiu brewing.

## Figures and Tables

**Table 1:** The impact of different protein components on the oenological parameters of millet Huangjiu.

	CK	Glu-1	Glu-2	Alb-1	Alb-2	Glu + Alb
Total soluble solids (°Bx)	11.80 ± 0.19 d	14.10 ± 0.33 b	15.6 ± 0.20 a	13.2 ± 0.11 c	12.5 ± 0.08 d	14.6 ± 0.41 b
Amino acid nitrogen (g/L)	0.65 ± 0.04 d	0.85 ± 0.03 b	0.92 ± 0.05 a	0.72 ± 0.02 c	0.76 ± 0.06 c	0.87 ± 0.07 b
Reducing sugar content (g/L)	7.62 ± 0.15 d	8.89 ± 0.23 c	9.54 ± 0.28 b	10.10 ± 0.17 a	8.15 ± 0.33 c	8.43 ± 0.26 c
Ethanol (% vol)	12.40 ± 0.11 c	13.50 ± 0.08 b	14.60 ± 0.16 a	12.80 ± 0.27 c	12.50 ± 0.04 c	13.30 ± 0.22 b

Values are presented as means ± standard deviations. Different letters in the same row indicate significant differences (*p* < 0.05).

**Table 2:** The impact of different protein components on the free amino acid fraction of millet Huangjiu.

Amino Acids (mg/L)	CK	Glu-1	Glu-2	Alb-1	Alb-2	Glu + Alb
**Umami amino acids**						
Aspartic acid	90.4 ± 5.4 d	107.9 ± 2.2 b	135.4 ± 5.9 a	90.7 ± 0.5 d	92.1 ± 1.2 d	100.2 ± 3.0 c
Glutamic acid	268.2 ± 15.9 c	299.7 ± 12.2 b	332.3 ± 18.7 a	283.1 ± 31.4 c	290.3 ± 23.8 c	310.0 ± 8.7 b
**Sweet amino acids**						
Serine	75.3 ± 7.6 c	67.2 ± 0.2 c	79.8 ± 0.4 c	85.1 ± 4.5 ab	89.2 ± 0.4 a	82.9 ± 0.2 b
Glycine	165.3 ± 2.4 c	174.7 ± 8.5 c	160.5 ± 3.3 c	181.6 ± 6.8 b	197.6 ± 0.8 a	193.4 ± 2.8 a
Threonine	93.1 ± 4.6 c	96.0 ± 1.6 c	91.7 ± 1.1 c	102.9 ± 2.2 ab	112.8 ± 0.6 a	110.9 ± 1.2 a
Alanine	195.6 ± 11.1 c	363.2 ± 20.2 a	381.2 ± 19.0 a	209.2 ± 22.3 c	251.3 ± 2.9 b	351.7 ± 6.3 a
Proline	155.5 ± 9.3 d	204.2 ± 15.0 c	240.7 ± 9.7 b	231.4 ± 9.8 bc	275.6 ± 5.0 a	217.9 ± 4.6 c
Lysine	53.2 ± 0.9 e	63.3 ± 0.4 d	82.2 ± 1.0 b	87.4 ± 3.0 a	71.6 ± 1.3 c	72.3 ± 1.9 c
**Bitter amino acids**						
Histidine	25.8 ± 3.2 b	28.3 ± 0.3 ab	29.1 ± 0.4 a	22.9 ± 0.2 c	24.7 ± 0.7 bc	21.6 ± 0.9 c
Arginine	45.1 ± 2.4 c	54.0 ± 10.4 bc	99.7 ± 0.8 a	49.0 ± 5.5 c	46.6 ± 3.1 c	58.1 ± 6.8 b
Tyrosine	61.2 ± 3.3 c	77.3 ± 2.7 bc	87.1 ± 1.2 a	81.4 ± 2.7 a	89.3 ± 1.6 a	85.7 ± 3.3 a
Valine	55.5 ± 1.7 e	67.9 ± 2.9 c	95.9 ± 1.2 a	62.6 ± 2.3 d	60.4 ± 1.1 d	81.4 ± 1.3 b
Methionine	43.3 ± 3.6 d	40.8 ± 0.2 d	51.6 ± 0.2 c	35.9 ± 0.5 e	58.0 ± 0.1 b	75.6 ± 0.4 a
Isoleucine	89.9 ± 5.8 d	98.6 ± 5.1 c	178.4 ± 0.2 a	90.7 ± 1.0 d	98.4 ± 8.4 cd	125.9 ± 1.4 b
Leucine	131.4 ± 28.6 bc	154.8 ± 3.1 b	297.0 ± 0.5 a	100.0 ± 1.9 d	127.2 ± 2.5 c	165.6 ± 8.2 b
Cysteine	36.8 ± 0.9 a	27.9 ± 0.7 a	31.2 ± 0.1 a	36.7 ± 0.8 a	44.0 ± 7.2 a	37.4 ± 0.1 a
Phenylalanine	127.1 ± 11.6 e	202.1 ± 2.8 f	193.6 ± 1.0 a	85.7 ± 2.3 d	89.1 ± 1.4 c	171.7 ± 2.7 b
Total	1712.7 ± 33.4 e	2127.9 ± 40.8 c	2567.4 ± 49.6 a	1836.3 ± 27.7 d	2018.2 ± 50.1 c	2262.3 ± 20.2 b

Values are presented as means ± standard deviations. Different letters in the same row indicate significant differences (*p* < 0.05).

**Table 3:** Identification of volatile organic compounds (VOCs) in millet Huangjiu.

Compound	RI (ref) ^a^	RI (cal) ^b^	CAS	Odor Descriptor	Identification ^c^
***Alcohols***					
2-Methyl-1-propanol	1101	1092	78-83-1	Malty	MS, RI
3-Methyl-1-butanol	1209	1212	123-51-3	Malty	MS, RI
1-Hexanol	1363	1356	111-27-3	Grassy, marzipan-like	MS, RI
1-Octen-3-ol	1430	1456	3391-86-4	Mushroom-like	MS, RI
1-Heptanol	1447	1465	111-70-6	Fruity, soapy	MS, RI
5-Methyl-1-hexanol	1466	1470	627-98-5	Fruity, soapy	MS, RI
1-Octanol	1573	1561	111-87-5	Soapy, citrus-like, green	MS, RI
2-Furanmethanol	1635	1656	98-00-0	Burnt, sweet	MS, RI
2-Octen-1-ol	1645	1628	22104-78-5	Soapy	MS, RI
1-Nonanol	1666	1640	143-08-8	Soapy, fruity	MS, RI
1-Decanol	1771	1763	112-30-1	Soapy	MS, RI
2-Butyl-1-octanol	1853	1849	3913-02-8	-	MS, RI
2-Phenylethanol	1902	1873	60-12-8	Floral, honey-like	MS, RI
***Esters***					
Ethyl acetate	887	880	141-78-6	Fruity, sweet	MS, RI
Ethyl butanoate	1026	1044	105-54-4	-	MS, RI
3-Methylbutyl acetate	1132	1121	123-92-2	Banana-like, fruity	MS, RI
Pentyl acetate	1175	1181	628-63-7	Pear, overripe banana	MS, RI
Ethyl hexanoate	1241	1232	123-66-0	Fruity, pineapple-like	MS, RI
Ethyl lactate	1341	1352	97-64-3	Fruity, pineapple	MS, RI
Ethyl heptanoate	1352	1341	106-30-9	Banana, strawberry	MS, RI
Butyl hexanoate	1407	1421	626-82-4	Fruity, pineapple	MS, RI
Ethyl octanoate	1441	1435	106-32-1	Pineapple, mushroom	MS, RI
Ethyl nonanoate	1526	1541	123-29-5	Waxy, soapy, grape	MS, RI
Isobornyl acetate	1582	1552	125-12-2	Herbal, citrus nuance	MS, RI
Isoamyl lactate	1619	1603	19329-89-6	Fruity, creamy, nutty	MS, RI
Ethyl decanoate	1648	1633	110-38-3	Soapy, pear-like	MS, RI
Diethyl succinate	1687	1669	123-25-1	-	MS, RI
Phenethyl acetate	1820	1813	103-45-7	Sweet, honey, floral	MS, RI
Ethyl dodecanoate	1847	1856	106-33-2	Waxy, soapy	MS, RI
γ-Nonanolactone	2020	2010	104-61-0	Coconut, creamy	MS, RI
Ethyl tetradecanoate	2070	2059	124-06-1	Sweet, creamy	MS, RI
Ethyl pentadecanoate	2161	2179	41114-00-5	Honey sweet	MS, RI
Ethyl hexadecanoate	2270	2288	628-97-7	Waxy, fruity, creamy	MS, RI
Ethyl oleate	2476	2452	111-62-6	Fatty, buttery	MS, RI
Ethyl linoleate	2510	2521	544-35-4	Fatty, fruity, oily	MS, RI
***Aldehydes***					
Hexanal	1083	1079	66-25-1	Green, grassy	MS, RI
Heptanal	1186	1180	111-71-7	Citrus-like, fatty	MS, RI
(E)-2-Heptenal	1334	1338	18829-55-5	Green-apple-like	MS, RI
Nonanal	1382	1379	124-19-6	Citrus-like, soapy	MS, RI
(E)-2-Octenal	1425	1430	2548-87-0	Fatty, nutty	MS, RI
Furfural	1470	1474	98-01-1	Sweet, cereal-like	MS, RI
Decanal	1486	1485	112-31-2	Fatty, orange peel	MS, RI
Benzaldehyde	1533	1525	100-52-7	Bitter-almond-like,	MS, RI
Undecanal	1593	1611	112-44-7	-	MS, RI
4-Methyl- benzaldehyde	1653	1643	104-87-0	-	MS, RI
***Acids***					
Acetic acid	1445	1451	64-19-7	Vinegar-like	MS, RI
Pentanoic acid	1736	1750	109-52-4	Acidic, milky, cheese	MS, RI
Hexanoic acid	1861	1854	142-62-1	Sweaty	MS, RI
Octanoic acid	2082	2075	124-07-2	Carrot-like, musty	MS, RI
nonanoic acid	2164	2176	112-05-0	Moldy, pungent	MS, RI
Decanoic acid	2268	2284	334-48-5	Soapy, musty	MS, RI
***Ketones***					
2-Octanone	1297	1283	111-13-7	Soapy, fruity	MS, RI
2-Nonanone	1403	1389	821-55-6	Fruity, musty	MS, RI
***Phenols***					
2-Methoxy-phenol	1865	1837	90-05-1	-	MS, RI
4-ethyl-2-methoxyphenol	2048	2054	2785-89-9	Smoky, gammon-like	MS, RI
2-methoxy-4-vinylphenol	2180	2184	7786-61-0	Smoky, clove-like	MS, RI

^a^ RI (ref), the Kovats retention index information obtained from the NIST Chemistry WebBook database (https://webbook.nist.gov/chemistry/name-ser/ (accessed on 5 January 2023)). ^b^ RI (cal), the experimental Kovats retention index calculated based on a DB-WAX capillary column. ^c^ Identification based on the MS (mass spectrum) and KI (Kovats retention index) information.

## Data Availability

The data is not to be shared for the moment.
